# *Aggregatibacter actinomycetemcomitans* mediates protection of *Porphyromonas gingivalis* from *Streptococcus sanguinis* hydrogen peroxide production in multi-species biofilms

**DOI:** 10.1038/s41598-019-41467-9

**Published:** 2019-03-20

**Authors:** Bin Zhu, Lorna C. Macleod, Eric Newsome, Jinlin Liu, Ping Xu

**Affiliations:** 1 Philips Institute for Oral Health Research, Virginia Commonwealth University, Richmond, VA 23298 United States of America; 2 College of Life Sciences, Central China Normal University, Wuhan, Hubei 430079 China; 3 Microbiology and Immunology Department, Virginia Commonwealth University, Richmond, VA 23298 United States of America; 4 Center for Biological Data Science, Virginia Commonwealth University, Richmond, VA 23298 United States of America

## Abstract

Mixed species biofilms are shaped and influenced by interactions between species. In the oral cavity, dysbiosis of the microbiome leads to diseases such as periodontitis. *Porphyromonas gingivalis* is a keystone pathogen of periodontitis. In this study, we showed that polymicrobial biofilm formation promoted the tolerance of *Porphyromonas gingivalis* to oxidative stress under micro-aerobic conditions. The presence of *Streptococcus sanguinis*, an oral commensal bacterium, inhibited the survival of *P. gingivalis* in dual-species biofilms via the secretion of hydrogen peroxide (H_2_O_2_). Interestingly, this repression could be attenuated by the presence of *Aggregatibacter actinomycetemcomitans* in tri-species biofilms. It was also shown that the *katA* gene, encoding a cytoplasmic catalase in *A. actinomycetemcomitans*, was responsible for the reduction of H_2_O_2_ produced by *S. sanguinis*, which consequently increased the biomass of *P. gingivalis* in tri-species biofilms. Collectively, these findings reveal that polymicrobial interactions play important roles in shaping bacterial community in biofilm. The existence of catalase producers may support the colonization of pathogens vulnerable to H_2_O_2_, in the oral cavity. The catalase may be a potential drug target to aid in the prevention of periodontitis.

## Introduction

According to the 2016 global burden of disease study, periodontal disease is estimated to affect 750,847 million people worldwide, making it the 11^th^ most prevalent human disease^1^. It is an inflammatory disorder, characterized by the destruction of tooth-supporting tissues such as gingiva, periodontal ligament and alveolar bone. Periodontitis is caused by the dysbiosis of the oral microbiome. In the pathogenesis of periodontitis, the subgingival microbiome switches from majority Gram-positive to majority Gram-negative bacterial species^2^.

*Porphyromonas gingivalis* is a Gram-negative bacterium which is regarded as one of the keystone pathogens in chronic periodontitis^3,4^. It produces virulence factors to disrupt host–microbial homeostasis, resulting in inflammation and bone loss^5,6^. Oral microbiome studies by 16s rRNA sequencing suggest that the abundance of periodontitis-associated species, such as *P. gingivalis*, *Treponema denticola* and *Tannerella forsythia*, is significantly increased in disease sites of periodontitis patients^7,8^. Many of these pathogens are anaerobic species that survive in deep dental pockets where oxygen is limited. Surprisingly, these anaerobic pathogens, including *P. gingivalis*, have also been reported in supragingival plaque, saliva and mucosa samples^9,10^, which are thought to be more micro-aerobic environments in the oral cavity. Several proteins have been reported to participate in the resistance to oxidative stress in *P. gingivalis*^11–14^ and they may promote the survival of *P. gingivalis* under micro-aerobic conditions.

The oxidative stress in the oral microbiome is not only related to oxygen concentration in the surroundings but reactive oxygen species (ROS) produced by eukaryotic cells and some oral commensal bacteria^15–17^. ROS leads to protein, DNA, and lipid damage, and results in an increased rate of mutagenesis and cell death^18^. *Streptococcus sanguinis* is a Gram-positive, facultative anaerobe bacterium that is able to inhibit the growth of *P. gingivalis*^15^ and produce a ROS, hydrogen peroxide (H_2_O_2_)^19,20^. It is a pioneering colonizer in the oral cavity and a key player in oral biofilm development^21,22^. Because the abundance was significantly decreased in the diseased subgingival microbiome, *S. sanguinis* was thought to be an oral health-associated species^7,8^. It is feasible that *S. sanguinis* maintains a healthy oral homeostasis by generating H_2_O_2_ in the oral cavity. Several genes have been reported to be responsible for H_2_O_2_ production in *S. sanguinis* SK36^19,20^.

*P. gingivalis* living in the oral cavity, particularly in sites under micro-aerobic conditions, must endure oxidative stress from the H_2_O_2_ produced by *S. sanguinis*. What are the mechanisms by which *P. gingivalis* tolerates oxidative stress under micro-aerobic conditions in oral microbiota?

*Aggregatibacter actinomycetemcomitans* is a Gram-negative, facultative anaerobe bacterium that is often found in chronic periodontitis^23^. The *katA* gene in *A. actinomycetemcomitans* encodes a cytoplasmic catalase that breaks down H_2_O_2_^24^. When *A. actinomycetemcomitans* was co-cultured with *Streptococcus gordonii*, the expression of the *katA* gene was induced by the H_2_O_2_ released from *S. gordonii*. This regulation was mediated via the upstream regulator OxyR^24^. Both *oxyR* and *katA* were important for the survival of *A. actinomycetemcomitans* in the presence of *S. gordonii*^24,25^. Additionally, *katA* expression was higher in the biofilm state than in its planktonic state^24^. As *A. actinomycetemcomitans* can degrade H_2_O_2_, we proposed that *A. actinomycetemcomitans* might also confer protection to *P. gingivalis* from the damage of H_2_O_2_ produced by *S. sanguinis* in mixed species biofilm.

*S. gordonii*, another commensal bacterium and H_2_O_2_ producer in the oral cavity, is a well-studied species that interacts with *P. gingivalis*, though few papers discussed the interaction between *S. sanguinis* and *P. gingivalis*. FimA and Mfa1 fimbrial proteins mediate the attachment of *P. gingivalis* to *S. gordonii*^26,27^. Additionally, Mfa1 binds to streptococcal SspB protein^28–30^. The coadhesion between *P. gingivalis* and *S. gordonii* improves the biofilm formation of *P. gingivalis* on streptococcal substrates^31^. Moreover, *S. gordonii* generates streptococcal 4-aminobenzoate/para-amino benzoic acid (pABA), used for folate biosynthesis, that results in decreased stress and promotes expression of fimbrial adhesins in *P. gingivalis*^32^. These studies show that *S. gordonii* supports the biofilm formation of *P. gingivalis*. However, most of these studies were performed under anaerobic conditions. The generation of H_2_O_2_ may be limited under these conditions. Thus, the killing effect of *P. gingivalis* by *S. gordonii* may be attenuated under anaerobic conditions when compared with micro-aerobic conditions.

In this study, we showed that *S. sanguinis* inhibited the growth of *P. gingivalis* by producing H_2_O_2_ under micro-aerobic conditions. *A. actinomycetemcomitans* reduced the concentration of H_2_O_2_ and consequently aided the survival of *P. gingivalis* in *S. sanguinis*-*P. gingivalis*-*A. actinomycetemcomitans* tri-species biofilms.

## Results

### The impact of *S. sanguinis* and *A. actinomycetemcomitans* on the biomass of *P. gingivalis* in multi-species biofilms

**Figure 1:**
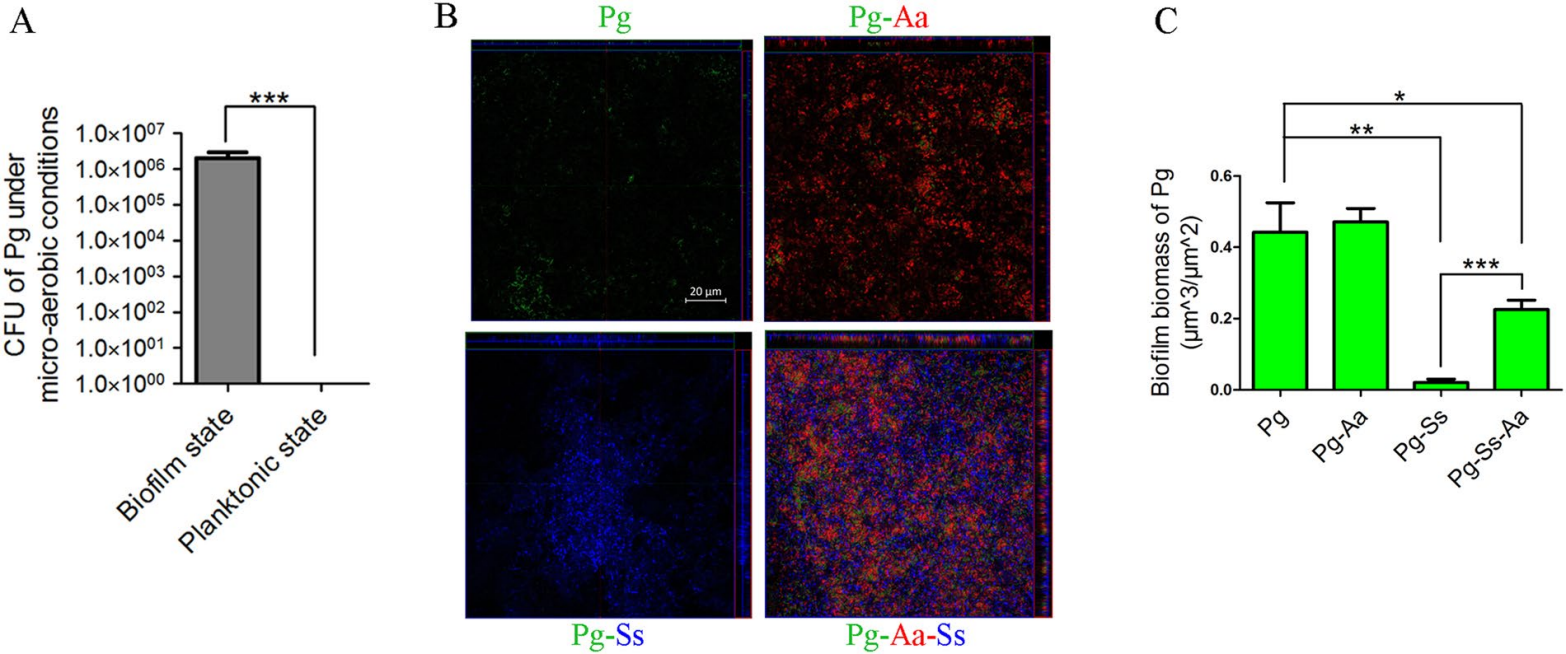
Confocal microscopical analysis of *Pg* biomass in 4-day old mixed-species biofilms under micro-aerobic conditions (6% O_2_). (**A**) *Pg* was incubated under static conditions for biofilm growth (left bar) and under shaking conditions (100 rpm) to prevent biofilm formation and to maintain a planktonic state (right bar is absent due to lack of growth). After 4 days, the CFUs from both samples were tested. (**B**) Single and multiplex staining of *Pg* (green), *Aa* (red) and *Ss* (blue) with FISH probes specific to the conserved 16s ribosomal (rRNA) genes of these bacteria (scale bar shown in top left panel = 20 µm). (**C**) *Pg* biomass in (**B**) was calculated for single and multiplex biofilms. *P ≤ 0.05, **P ≤ 0.01, ***P ≤ 0.001, Student’s *t-*test. Means and standard deviations from triplicate experiments are shown.

It has been reported that many anaerobic species, including *P. gingivalis*, are widely distributed in the oral cavity^9,10^. We performed experiments to evaluate the survival of *P. gingivalis* in 3 environments: 14 mL test tubes with shaking at 100 rpm under micro-aerobic conditions (6% oxygen, gas mixture), and 4-well chambers without shaking (static) under either anaerobic (0% oxygen, gas mixture) conditions or micro-aerobic conditions. The incubator shaking was used to inhibit biofilm growth and thus encourage planktonic growth while the lack of shaking was used to facilitate biofilm formation. All three environments had an initial inoculation of 1 × 10^8^ *P. gingivalis* ATCC 33277 (*Pg*) cells into CDM and were incubated for four days at 37 °C. When *Pg* was cultured in the 14 mL tubes environment, it was not able to survive (Fig. 1A). *Pg* survived in both 4-well chamber environments; the biomass under micro-aerobic conditions was significantly lower than that under anaerobic conditions (P ≤ 0.01) (Fig. S1). *Pg* cells from the micro-aerobic 4-well chamber were then inoculated onto agar plates and grown in order to verify that the detected fluorescent signal was from live cells. The growth of 2.03 × 10^6^ ± 9.71 × 10^5^ colony-forming units (CFUs) confirmed the existence of live biofilm cells in static micro-aerobic conditions (Fig. 1A). Although the CFU of *Pg* grown under micro-aerobic conditions in 4-well chambers was lower than that of the initial inoculation, it was still greater than the CFU of *Pg* grown in the 14 mL test tube, suggesting that the biofilm formation increased the tolerance of *Pg* to oxidative stress from the presence of environmental oxygen (Fig. 1A). Because there have already been a number of reports illustrating that biofilm formation increase the tolerance of bacteria to oxidative stress^33^, the next experiment was designed to focus on the effect of mixed-species biofilm bacterial interactions on the oxidative stress tolerance of *P. gingivalis*.

To examine *Pg* survival in multi-species biofilms, four groups of bacterial mixes were tested: *Pg* only, *Pg* and *A. actinomycetemcomitans* 652 (*Aa*), *Pg* and *S. sanguinis* SK36 (*Ss*) and a mixture of all three species. Each bacterial mix group was incubated in CDM medium under micro-aerobic conditions in 4-well chambers. Biofilms were first grown for four days, after which they were stained by fluorescence *in situ* hybridization (FISH) and were visualized using confocal laser scanning microscopy (CLSM). Biofilm biomass was quantified by COMSTAT script in Matlab software^33^.

All four groups of biofilms were successfully detected using FISH probes. Under these micro-aerobic conditions, the biomass of *Pg* in biofilm was not significantly changed by the presence of *Aa* when compared to the *Pg* single species biofilm control (Fig. 1B,C). In contrast, the presence of *Ss* significantly lowered the biomass of *Pg* in *Ss-Pg* dual-species biofilms, suggesting that *Ss* was dominant and somehow inhibited survival of *Pg* (0.442 ± 0.083 µm^3^/µm^2^ in *Pg* only biofilm and 0.021 ± 0.009 µm^3^/µm^2^ in *Pg-Ss* dual species biofilm (P ≤ 0.001)) (Fig. 1B,C). Interestingly, the biomass of *Pg* in *Pg*-*Aa*-*Ss* tri-species biofilms was significantly increased compared to that in *Pg*-*Ss* dual-species biofilms, implying that *Pg* survival inhibition by *Ss* could be partially attenuated by the presence of *Aa* (Fig. 1B,C). As there was no significant difference between the biomass of *Pg* from *Pg* single species biofilms and *Pg*-*Aa* dual-species biofilms, it is feasible to suggest that *Aa* could interact with *Ss*, counteracting the influence of *Ss* on *Pg*, indirectly promoting the survival of *Pg*.

### *Ss*-produced H_2_O_2_ reduced the biomass of *Pg*

**Figure 2:**
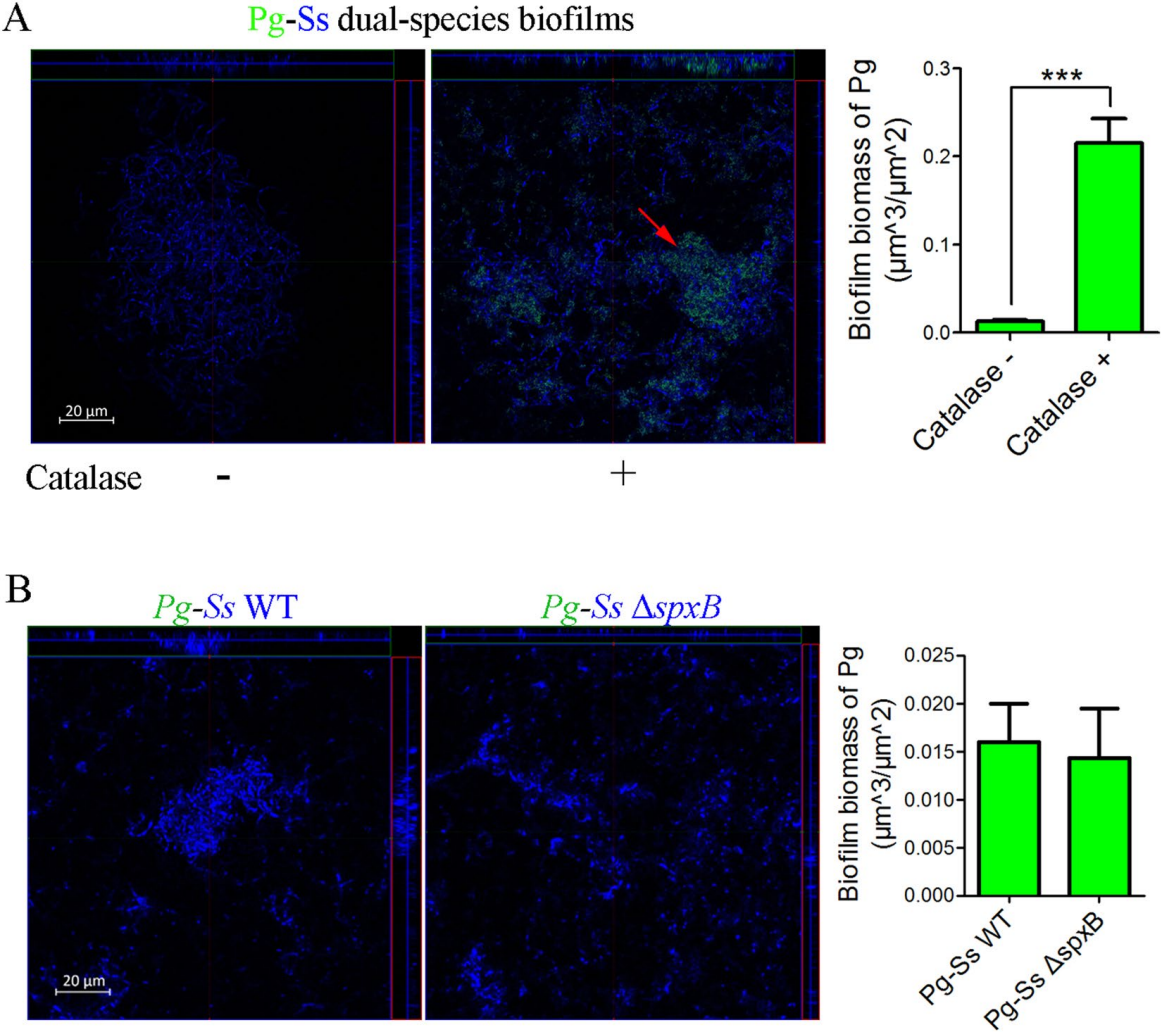
Confocal microscopical images of 4-day old *Pg-Ss* dual-species biofilms to analyze the effect of H_2_O_2_ on *Pg* biomass under micro-aerobic conditions. (**A**) *Pg-Ss* dual-species biofilms (*Pg* = green, *Ss* = blue) with/without the treatment of 10,000 U/mL of catalase. (**B**) *Pg-Ss* wild type (WT) and *Pg-Ss* Δ*spxB* dual-species biofilms. Samples were stained by FISH probes. Orthogonal CLSM images were shown in the left panel. Scale bars were indicated on the corresponding images. In the right panel, the biomass of *Pg* from images in the left panel was quantified by COMSTAT analysis. ***P ≤ 0.001, Student’s *t-*test. Means and standard deviations from triplicate experiments are shown.

H_2_O_2_ is a well-studied inhibitory mechanism that *S. sanguinis* uses to compete with *Streptococcus mutans*^19,34^. It can be generated by a pyruvate oxidase (SpxB) in *Ss* via a reaction converting pyruvate to acetyl phosphate. During this catalytic process, oxygen is consumed^35,36^. *Pg-Ss* dual-species biofilms were grown under micro-aerobic conditions. We tested the inhibitory ability of *Ss*-produced H_2_O_2_ by decomposing H_2_O_2_ with 10,000 U/mL of catalase (Catalase from bovine liver, Sigma). More *Pg* was present in the dual-species biofilms when catalase was supplemented in the medium (P ≤ 0.001) (Fig. 2A). This result suggested that H_2_O_2_ was essential for *Ss* to inhibit the survival of *Pg* under micro-aerobic conditions. *Pg* appeared to preferentially colocalize with *Ss* in *Pg-Ss* dual-species biofilms when catalase was supplemented. This phenomenon implied that *Pg* might coaggregate with *Ss* in multi-species biofilms, which was similar to the interaction between *P. gingivalis* and *S. gordonii*^29^.

When *Pg* was co-cultured with *Ss* Δ*spxB*, the biomass of *Pg* was still greatly inhibited by *Ss* Δ*spxB* (Fig. 2B). One probability was that, in contrast with *Ss* wild type (WT), the Δ*spxB* mutant could still produce about 25% the concentration of H_2_O_2_^19^, which might be enough for the inhibition of *Pg* growth.

**Figure 3:**
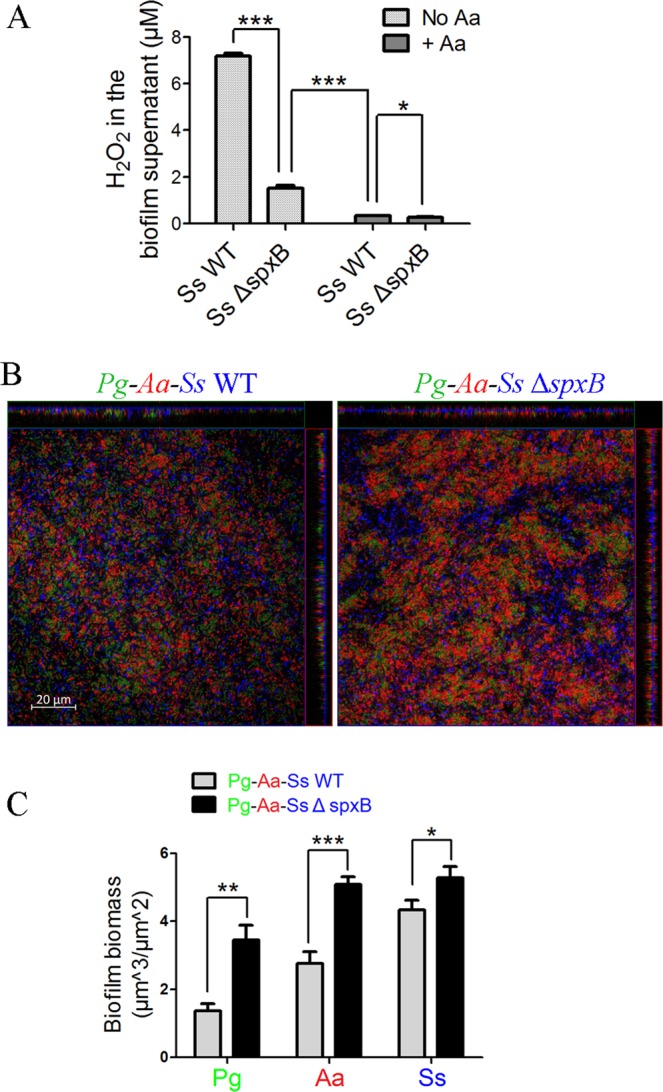
The effect of the *spxB* gene deletion on *Pg* biomass in *Pg-Aa-Ss* tri-species biofilms under micro-aerobic conditions. (**A**) *Ss* WT and *Ss* Δ*spxB* biofilms with/without the supplement of *Aa* were cultured. The H_2_O_2_ concentration in the supernatant of these biofilms was measured by the Hydrogen Peroxide Assay as described in Materials and methods. (**B**) *Pg*-*Aa*-*Ss* WT (left) and *Pg*-*Aa-Ss* Δ*spxB* tri-species (right) (*Pg* = green, *Ss* = blue, *Aa* = red) biofilms were shown. (**C**) The biomass of *Pg*, *Aa* and *Ss* in **B** was quantified by COMSTAT and shown as a bar chart. Scale bars were indicated on the corresponding images. *P ≤ 0.05, **P ≤ 0.01, ***P ≤ 0.001, Student’s *t-*test. Means and standard deviations from triplicate experiments are shown.

Since more *Pg* survived in the *Pg-Aa-Ss* tri-species biofilm, the supplementation of *Aa* might have a better effect than the deletion of *spxB* on reducing H_2_O_2_ concentration. To test this hypothesis, *Ss* WT and *Ss* Δ*spxB* biofilms with/without the addition of *Aa* were cultured. The amount of H_2_O_2_ in the supernatant of the 4-day old biofilms was measured using Hydrogen Peroxide Assay. Indeed, the H_2_O_2_ concentration in the *Aa-Ss* WT dual-species biofilm was much lower than that in the *Ss* Δ*spxB* single species biofilm (P ≤ 0.0001), which supported the hypothesis (Fig. 3A). Additionally, in comparison to the *Ss* WT single species biofilm, the *Ss* Δ*spxB* single species biofilm contained less H_2_O_2_ in the supernatant (P ≤ 0.0001), which was consistent with the result in the previous study showing that the *spxB* gene deletion decreased H_2_O_2_ production in *Ss* (Fig. 3A)^19^.

The *Aa*-*Ss* Δ*spxB* dual-species biofilm contained less H_2_O_2_ than the *Aa-Ss* WT biofilm, which indicated that the *spxB* gene deletion might promote *Pg* survival in *Pg-Aa-Ss* tri-species biofilms (Fig. 3A). 4-day old *Pg*-*Aa*-*Ss* WT and *Pg*-*Aa*-*Ss* Δ*spxB* tri-species biofilms were treated with FISH and observed by CLSM. The biomass of *Pg* in the *Pg*-*Aa*-*Ss* Δ*spxB* tri-species biofilm was more than that in the *Pg*-*Aa*-*Ss* WT biofilm (P ≤ 0.01), which suggested that the H_2_O_2_ produced by *Ss* played an important role in inhibiting *Pg* growth (Fig. 3B,C).

Similar to *Pg*, the biomass of *Aa* was also increased in the *Pg*-*Aa*-*Ss* Δ*spxB* biofilm (P ≤ 0.001) (Fig. 3B,C). Surprisingly, the biofilm biomass of *Ss* was increased in the *Pg*-*Aa*-*Ss* Δ*spxB* biofilms than that in the *Pg*-*Aa*-*Ss* WT tri-species biofilm (P ≤ 0.05) (Fig. 3B,C), despite *Ss* Δ*spxB* had a reduced biofilm formation in *Ss-Pg* dual-species biofilms (Figs 2B and S2). When we treated tri-species biofilms using FISH protocol, we observed that the *Pg*-*Aa*-*Ss* WT biofilm was more fragile than the *Pg*-*Aa*-*Ss* Δ*spxB* biofilm, indicating that H_2_O_2_ might affect inter-species attachment. It has been shown that *P. gingivalis* utilizes fimbrillin to bind to glyceraldehyde-3-phosphate dehydrogenase, a cell surface protein of *S. sanguinis*^37^, implying that *S. sanguinis* may not only inhibit the growth of *P. gingivalis* but also coaggregate with *P. gingivalis*. Due to the deletion of *spxB*, a reduced antagonism in *Pg-Aa-Ss* tri-species biofilm might be beneficial for the co-aggregation between *Pg* and *Ss*, and as a result, might increase the biomass of *Ss*. Though a similar relationship may exist between *S. sanguinis* and *A. actinomycetemcomitans*, the current knowledge on their interactions is limited, and the mechanism of such phenomenon needs further exploration.

### *Aa* degraded H_2_O_2_ and protected *Pg* from H_2_O_2_ attack

The data in Fig. 3A implied that *Aa* might impact *Ss* and indirectly promoted the survival of *Pg* through degrading H_2_O_2_. To further elucidate whether *Aa* protected *Pg* from H_2_O_2_, the concentration of H_2_O_2_ was measured using Hydrogen Peroxide Assay. Briefly, cells were resuspended in fresh CDM and mixed with Hydrogen Peroxide Assay solution. The final reaction solutions were incubated under room atmospheric conditions at 37 °C. The optical density for cell growth and fluorescent signal for H_2_O_2_ concentration were monitored by the plate reader.

**Figure 4:**
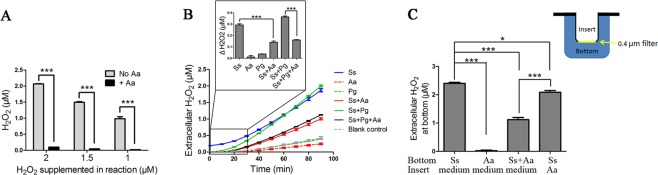
The effect of *Aa* on the concentration of H_2_O_2_. (**A**) *Aa* suspensions were mixed with H_2_O_2_ solutions. H_2_O_2_ concentrations were then tested after 30 minutes of reaction. (**B**) H_2_O_2_ concentrations of different bacterial mixtures were measured at 10-minute intervals. The enlarged section of the graph, illustrated as a bar chart, showed the H_2_O_2_ concentration after 30 minutes of reaction. CDM media without bacteria was used as a blank control. (**C**) Wells were divided into two parts by a transwell system. *Ss*, *Aa* or CDM medium were located at different places as shown in the figure. The H_2_O_2_ concentration in the bottom section was measured. *P ≤ 0.05, ***P ≤ 0.001, Student’s *t-*test. Means and standard deviations from triplicate experiments are shown.

Firstly, the Hydrogen Peroxide Assay solution was supplemented with H_2_O_2_ (2, 1.5 or 1 μM). It was mixed or not mixed with *Aa* suspension. The H_2_O_2_ concentration was recorded after 30 minutes of reaction. The presence of *Aa* greatly reduced H_2_O_2_ concentrations for all variants of the Hydrogen Peroxide Assay solution, which demonstrated that *Aa* has the ability to degrade H_2_O_2_ (Fig. 4A).

Subsequently, the H_2_O_2_ produced by *Ss*, *Pg* and *Aa* was tested. Compared with the blank control (CDM without bacteria), neither *Pg* nor *Aa* produced H_2_O_2_ (Fig. 4B). The H_2_O_2_ concentration in *Aa* was even lower than the concentration in the blank control (Fig. 4B). *Ss* produced almost 0.3 μM of H_2_O_2_ after 30 minutes of reaction, which could be attenuated by the addition of *Aa* (P ≤ 0.001) but not *Pg* (Fig. 4B). The presence of *Aa* decreased the H_2_O_2_ concentration by nearly half in both the dual-species (*Ss*-*Aa*) and tri-species (SS-*Pg*-*Aa*) suspensions (Fig. 4B). There was no significant change in cell density during the 30 minutes of experimentation, indicating that cell growth did not influence the results of H_2_O_2_ concentrations (Fig. S3). Similar results were reported in previous works, which utilized scanning electrochemical microscopy to do real-time mapping of H_2_O_2_ concentrations on bacteria biofilms^38^. They reported that the H_2_O_2_ generated by *S. gordonii*, another oral commensal bacterium and H_2_O_2_ producer, could be reduced by *A. actinomycetemcomitans*^38^. These data suggested that *Aa* degraded H_2_O_2_ produced by *Ss* and implied that *Aa* might be able to promote the survival of *Pg* in *Pg*-*Aa*-*Ss* tri-species biofilm by reducing H_2_O_2_ concentration.

Bacteria living in biofilms are surrounded by matrix composed of polysaccharide, eDNA and proteins^39^. As materials may slow penetrate and transverse a biofilm^39^, cell to cell distance may impact the interaction between *Aa* and *Ss*. To test the contribution of cell-cell distance to the *Aa*–*Ss* interaction, *Aa* and *Ss* were cultured in a transwell system, where they were separated by a 0.4 μm filter. *Ss* was cultured at the bottom and *Aa* was either incubated in the insert or mixed with *Ss* at the bottom. After 30 minutes of reaction, the H_2_O_2_ concentration at the bottom of the well was measured. The H_2_O_2_ concentration at the bottom was 2.404 ± 0.035 μM when the insert was filled with CDM medium and the bottom was *Ss*. When *Aa* was put in the insert and *Ss* was set at the bottom of the well, *Aa* slightly but significantly decreased the H_2_O_2_ concentration at the bottom to 2.087 ± 0.061 μM (P ≤ 0.05) (Fig. 4C). However, the reduction was much lower than that in the well where *Ss* and *Aa* mixed directly at the bottom (P ≤ 0.001) (Fig. 4C). This result showed that a closer distance between *Ss* and *Aa* was beneficial for *Aa* to reduce H_2_O_2_ produced by *Ss*. Aa might have limited function to degrade H_2_O_2_ when it was far away from *Ss*. In an *in vitro* study, *Aggregatibacter* has been shown to close contact with *Streptococcus*^40^, indicating that *A. actinomycetemcomitans* might exist near to *S. sanguinis in vivo* to detoxify H_2_O_2_.

KatA has been reported to produce catalase in *A. actinomycetemcomitans* strain VT1169 (*Aa* VT1169) to detoxify H_2_O_2_ and is essential for the survival of *A. actinomycetemcomitans* during co-infection with *S. gordonii*^24,25^. It was hypothesized that KatA was essential for *Aa* to improve the survival of *Pg*.

**Figure 5:**
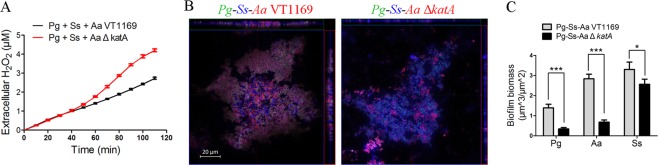
The influence of the *katA* gene of *Aa* VT1169 on *Pg*-*Ss* -*Aa* VT1169 tri-species biofilms. (**A**) The H_2_O_2_ concentrations of *Pg*-*Ss*-*Aa* VT1169 and *Pg*-*Ss*-*Aa* Δ*katA* were recorded at 10-minute intervals using the Hydrogen Peroxide Assay. (**B**) The biofilms of *Pg*-*Ss*-*Aa* VT1169 and *Pg*-*Ss*-*Aa* Δ*katA* were stained by FISH and observed by CLSM. *Pg*, *Aa* VT1169 and *Ss* were marked as green, red and blue, respectively. (**C**) The biomass of *Pg*, *Aa* VT1169 and *Ss* in (**B**) were quantified by COMSTAT. Scale bars were indicated on the corresponding images. *P ≤ 0.05, ***P ≤ 0.001, Student’s *t-*test. Means and standard deviations from triplicate experiments are shown.

Using the Hydrogen Peroxide Assay Kit, H_2_O_2_ concentration and cell density of *Pg* + *Ss* + *Aa* VT1169 and *Pg* + *Ss* + *A. actinomycetemcomitans* VT1169 Δ*katA* (*Aa* Δ*katA*) suspensions were monitored. Compared to the *katA* deletion mutant, *Aa* VT1169 had the greater ability to repress H_2_O_2_ production (P ≤ 0.001 at the time point of 110 minutes), implying that KatA was important for *Aa* to reduce the H_2_O_2_ generated by *Ss* (Fig. 5A). There was no significant difference in cell density between *Pg* + *Ss* + *Aa* VT1169 and *Pg* + *Ss* + *Aa* Δ*katA*, suggesting that the difference in H_2_O_2_ concentration was not caused by a difference in cell growth (Fig. S4).

*Pg*-*Ss*-*Aa* VT1169 and *Pg*-*Ss*-*Aa* Δ*katA* Tri-species biofilms were stained by FISH and observed by CLSM as described above. Compared with the biofilm of *Pg*-*Ss*-*Aa* VT1169, the *Pg*-*Ss*-*Aa* Δ*katA* biofilm contained less *Pg* and *Aa* (P ≤ 0.001 for both comparisons) (Fig. 5B,C), which suggested that the catalase of *A. actinomycetemcomitans* was essential for the survival of both *Pg* and *A. actinomycetemcomitans* in the tri-species biofilms and further confirmed the hypothesis that *A. actinomycetemcomitans* protected *P. gingivalis* from H_2_O_2_ damage. The biofilm biomass of *Ss* in *Pg*-*Ss*-*Aa* Δ*katA* was slightly decreased (P ≤ 0.05) (Fig. 5B,C). This phenomenon where *Ss* biofilm biomass decreased in conditions that also led to decreased biofilm biomass of *Aa* and *Pg*, was similar to the observed phenomenon in Fig. 3B,C allowing for the possible hypothesis that the biomass of *Ss* might have been impacted by *Pg* and/or *A. actinomycetemcomitans* in tri-species biofilms.

The VT1169 and Δ*katA* single species biofilms were stained by SYTO9 and observed by CLSM. The morphology of Δ*katA* biofilm was different from that of the wild type strain. The biofilm of Δ*katA* was much thicker (Fig. S5A). It contained larger aggregations and bigger gaps between aggregations (Fig. S5A). However, the biofilm biomass of these two strains were similar, which confirmed that the reduction of *Aa* biomass in *Pg*-*Ss*-*Aa* Δ*katA* tri-species was not caused by an attenuated biofilm formation ability of *Aa* Δ*katA* (Fig. S5).

## Discussion

In this study, it was shown that *Aa* degraded H_2_O_2_ produced by *Ss*, which consequently aided the survival of *Pg* in *Pg*-*Aa*-*Ss* tri-species biofilms under micro-aerobic conditions. KatA, which produces catalase in *Aa*, was also shown to participate in this interaction. There have been many epidemiological studies showing that anaerobic bacteria such as *P. gingivalis* exist in supragingival, salivary and mucosal samples^9,10^. One possibility is that micro-environment may exist in oral cavity, which allows these anaerobic bacteria to survive in micro-aerobic conditions. The results of this study presented the possibility that catalase producers in oral microbiota attenuate the oxidative stress and help the survival of anaerobic species under micro-aerobic conditions.

The pathogenesis of periodontitis has been thought to possess polymicrobial synergistic interactions^41,42^. A temporal dynamics study showed that facultative anaerobic bacteria, especially *Streptococcus*, were dominant in the early stage of oral biofilm formation^43^. Subsequently, the ‘healthy’ biofilm composition was replaced with a population of gram-negative anaerobic bacteria^43^. In our study, we observed essentially no *Pg* presence in *Pg*-*Ss* dual species biofilms but *Pg* presence was obvious in *Pg-Aa*-*Ss* tri-species biofilms, suggesting that the existence of *Aa* was important for *Pg* survival. This result indicated that the earlier colonization of bacteria species with catalase activity than anaerobic species in oral biofilms might be necessary to generate suitable surroundings for the survival of anaerobic microorganisms. Further studies need to be performed to test this hypothesis. Additionally, our study illustrated that KatA of *Aa* VT1169 was important for the growth of both *Pg* and *Aa* VT1169, implying that catalase might be a promising drug target to prevent periodontitis.

Welch *et al*. utilized FISH technology to stain supragingival dental plaque^40^. They hypothesized that the *Porphyromonas* growing at the periphery of biofilm samples might not be *P. gingivalis* because the outer shell of the biofilm was in a presumably aerobic environment^40^. Here, we demonstrated that *P. gingivalis* was able to survive in a micro-aerobic environment and had better survival in the presence of *A. actinomycetemcomitans*, which implied that it was possible that the bacteria at the periphery of supragingival biofilm samples, seen in the Welch’s study, was *P. gingivalis*. In their study, they showed that both *Porphyromonas* and *Haemophilus/Aggregatibacter* were in close contact with *Streptococcus* cells^40^. Furthermore, *Aggregatibacter* was not found adjacent to cells of *Porphyromonas* in the absence of *Streptococcus*^40^. Their results indicated that *P. gingivalis*, *A. actinomycetemcomitans* and *S. sanguinis* might be close to each other *in vivo* and a similar interaction between these three species might also exist *in vivo*.

In Fig. 2A, *Pg* appeared to preferentially colocalize with *Ss* in *Pg-Ss* dual-species biofilms when catalase was supplemented. Additionally, the biomass of *Ss* in both Figs 3B and 5B were positively related with the biomass of *Pg* and *Aa*. All the phenomena above indicated that *Ss* might also cooperate with *Pg* and/or *Aa* in multi-species biofilms. The antagonism and the cooperation between commensal bacteria and pathogens may exist in equilibrium in oral microbiota. Whenever the antagonism was weakened, or the cooperation was strengthened either by other microorganisms or environmental conditions, dysbiosis may happen and lead to diseases such as periodontitis.

## Materials and Methods

### Bacterial strains, growth and antibiotics

Strains used in this study are listed in Table S1. Unless otherwise stated, *Pg*, *A. actinomycetemcomitans* strains and *Ss* cells from −80 °C frozen glycerol stocks were 0.5% inoculated into TSB medium (tryptic soy broth supplemented with yeast extract (5 mg/ml), hemin (5 µg/ml) and menadione (1 µg/ml)) and incubated statically under anaerobic conditions (10% CO_2_, 10% H_2_ and 80% N_2_) at 37 °C using an Anoxomat^®^ system (Spiral Biotech, Norwood, MA). Spectinomycin was used at 50 μg/mL for the culture of *Aa* Δ*katA*. No antibiotic was added to multi-species biofilms. The CFUs of *Pg* were tested by growing *Pg* on sheep blood agar plates (Trypticase™ Soy Agar (TSA II™) with Sheep Blood, BD BBL™) under anaerobic conditions. All media was incubated in anaerobic jars for at least 2 days before experiments to equilibrate.

### Biofilm assay

*Pg*, *A. actinomycetemcomitans* strains and *Ss* were initially incubated separately for 48 hours, 24 hours and overnight respectively, in TSB medium under anaerobic conditions to early stationary phase. The resultant growth was then resuspended in fresh CDM, followed by 10% inoculation into CDM medium and incubation under micro-aerobic conditions for biofilm formation. CDM was prepared as previously described (10.0 mM of NaH_2_PO_4_, 10.0 mM of KCl, 2.0 mM of citric acid, 1.25 mM of MgCl_2_, 100 µM of FeCl_3_, 20.0 µM of CaCl_2_, 0.1 µM of Na_2_MoO_4_, 25.0 µM of ZnC1_2_, 50.0 µM of MnC1_2,_ 5.0 µM of CuCl_2,_ 10.0 µM of CoCl_2_, 5.0 µM of H_2_BO_3_, 1% (w/v) Tryptone, 7.67 µM of Hemin and 2.91 µM of Menadione)^44^. Biofilms were incubated in 4-chambered glass coverslip wells (Chambered Coverglass, Nunc™ Lab-Tek™) for 4 days at 37 °C. Cultures were grown anaerobically (0% O_2_, 10% CO_2_, 10% H_2_ and 80% N_2_) or micro-aerobically (6% O_2_, 7.2% CO_2_, 7.2% H_2_ and 79.6% N_2_) in jars using the Anoxomat^®^ system (Spiral Biotech, Norwood, MA). Single-species biofilms were stained using SYTO 9 (SYTO™ 9 Green Fluorescent Nucleic Acid Stain, Invitrogen™ Molecular Probes™) and FISH was used to analyze and characterize the composition in multi-species biofilms.

### FISH assay

FISH was performed as previously described^40^. FISH probes used in the study were ordered from Integrated DNA Technologies, Inc. and the sequences were listed in Table S2. Biofilms were grown in 4-well chambers for 4 days in 1 mL of CDM medium. 800 µL of supernatant was discarded by pipetting and then 4-well chambers were slowly turned over on paper towels to discard remaining supernatant. Biofilms were gently washed by 200 µL of 1× PBS buffer and fixed by 2% (wt/vol) paraformaldehyde on ice for at least 1.5 hours. After fixation, samples were gently washed again in 1× PBS for 15 min. Next, PBS was discarded and 10 µL of hybridization solution (900 mM of NaCl, 20 mM of Tris, pH 7.5, 0.01% of SDS, 20% (vol/vol) of formamide, each probe at a final concentration of 0.1 µM) was dropped on biofilm samples and stained at 46 °C for 4 hours in a chamber humidified with 20% (vol/vol) formamide. Samples were then gently washed in wash buffer (215 mM of NaCl, 20 mM of Tris, pH 7.5, 5 mM of EDTA) at 48 °C for 15 minutes. Finally, biofilms were gently washed by cold water, and mounted in ProLong Gold Antifade Solution (ThermoFisher) for CLSM observation.

### CLSM and biomass quantification

FISH-treated biofilms were observed by a Zeiss LSM710 confocal laser scanning microscope (Zeiss, Germany) (VCU Core Facilities) and quantified by COMSTAT in Matlab software^33^. The fluorescent dyes were listed in Table S2. Three images of each sample were quantified to calculate the means and standard deviations.

### Hydrogen Peroxide Assay

H_2_O_2_ concentration was measured by Hydrogen Peroxide Assay Kit (Red Hydrogen Peroxide/Peroxidase Assay Kit, Amplex™). Operations followed a standard protocol of the kit. For testing H_2_O_2_ concentration in biofilm supernatant, 80 ul of biofilm supernatant was centrifuged. Subsequently, 50 ul of supernatant was mixed with Hydrogen Peroxide Assay solution. After 30 minutes of reaction, the fluorescent signal (excitation 560 nm/emission 590 nm) was recorded by a Synergy H1 Hybrid Reader. The preparation of a standard curve for quantifying H_2_O_2_ concentration followed the standard protocol of the kit. To get data presented in Fig. 4B, *Pg*, *A. actinomycetemcomitans* strains and *Ss* were grown for 48 hours, 24 hours and overnight respectively in TSB to early stationary phase under anaerobic conditions. Cells were resuspended in fresh CDM and 10% inoculated into fresh CDM to get bacterial suspensions. 50 ul of bacteria suspensions were mixed with 50 ul of Hydrogen Peroxide Assay solution and incubated under laboratory atmospheric conditions at 37 °C using the Synergy H1 Hybrid Reader. The optical density (OD_600_) for cell growth and fluorescent signal for H_2_O_2_ concentration were monitored continuously by the reader. For testing H_2_O_2_ concentration in the transwell system (96 Well Permeable Support System transwell, Corning™ HTS Transwell™), *Aa* and *Ss* cells were grown to early stationary phase in TSB medium, followed by resuspension in fresh CDM. 50 ul of bacteria suspension and 50 ul of Hydrogen Peroxide Assay solution were mixed at the bottom of the well. The insert was filled with 50 ul of CDM or 50 ul of *Aa* suspension. After 30 minutes of reaction, the insert was discarded and the H_2_O_2_ concentration at the bottom of the well was measured. Three replicates were performed to calculate the means and standard deviations.

### Statistical analysis

All data were obtained from at least three biological replicates. Student’s t-test was applied to analyze data on biofilm biomass, H_2_O_2_ concentration and CFU.

## Supplementary information

Supporting information
