# Supplementary material for: Aggregatibacter actinomycetemcomitans mediates protection of Porphyromonas gingivalis from Streptococcus sanguinis hydrogen peroxide production in multi-species biofilms
Source: Sci Rep. 2019 Mar 20;9:4944. doi: 10.1038/s41598-019-41467-9 (PMC6426879; doi:10.1038/s41598-019-41467-9)
Supplement: Supplementary file 1 — Supporting information [file 41598_2019_41467_MOESM1_ESM.pdf]

***Aggregatibacter actinomycetemcomitans* mediates protection of *Porphyromonas gingivalis* from *Streptococcus sanguinis* hydrogen peroxide production in multi-species biofilms**

Bin Zhu[1], Lorna C Macleod[1], Eric Newsome[1], Jinlin Liu[2] and Ping Xu[1, 3, 4,*]

Supporting information

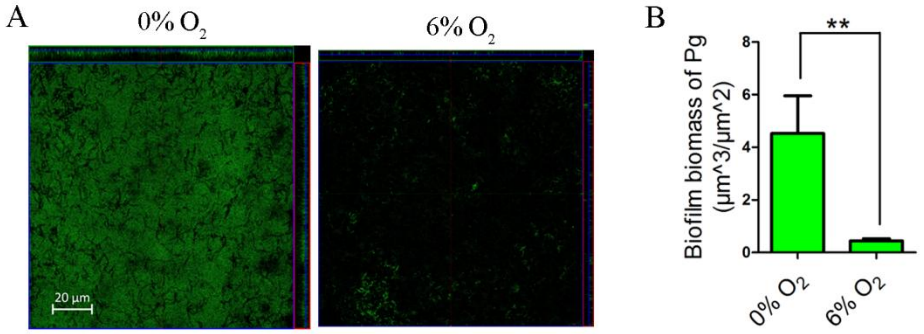

Fig. S1 CLSM analysis of *Pg* biomass in FISH stained 4 day-old biofilms grown under anaerobic and micro-aerobic conditions. (A) Orthogonal images of *Pg* biofilm grown in 0% O_2 (left) and 6% O_2 (right). Scale bar shown on left image = 20 µm. **(B)** The fluorescent signal from images in Fig. S1A was quantified by COMSTAT analysis. **P ≤ 0.01, Student's *t*-test. Means and standard deviations from triplicate experiments are shown.

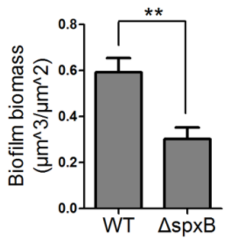

Fig. S2 CLSM analysis of *Ss* biomass in 4-day old FISH stained biofilms under micro-aerobic conditions. The biomass of *Ss* in figure 2B was quantified by COMSTAT and shown as a bar chart. Scale bars were indicated on the corresponding images. **P ≤ 0.01, Student's *t*-test. Means and standard deviations from triplicate experiments are shown.

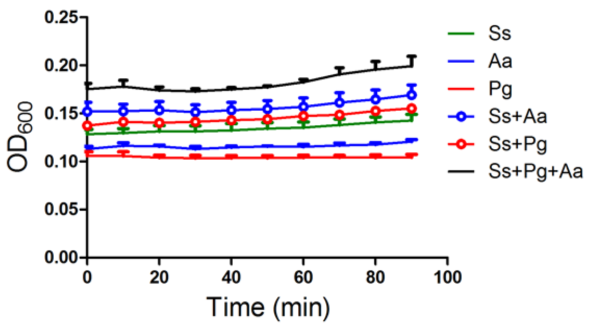

Fig. S3 Cell density measurements recorded during H_2O_2 concentrations time course experiment shown in Fig. 3B. Cell densities were recorded at intervals of 10 minutes using a Synergy H1 Hybrid Reader (OD_{600}).

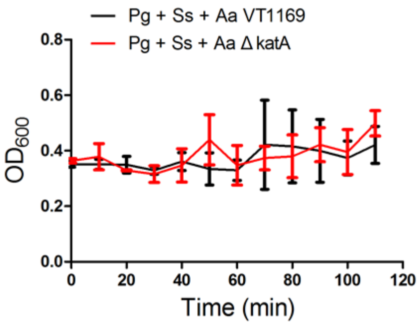

Fig. S4 Cell density measurements recorded during H_2O_2 concentrations time course experiment shown in Fig. 4A. Cell densities were recorded at intervals of 10 minutes using the Synergy H1 Hybrid Reader (OD_{600}).

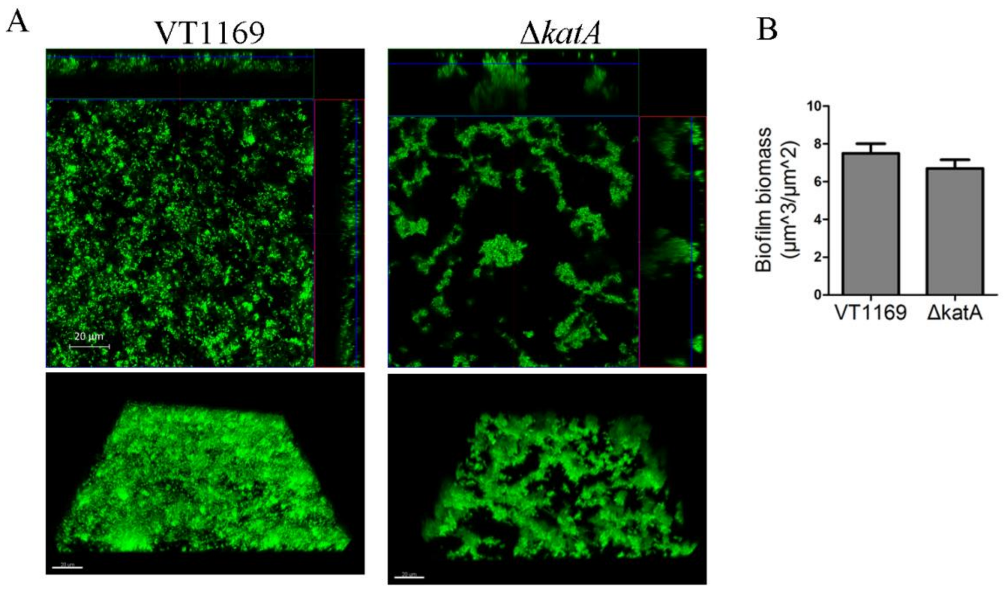

Fig. S5 The biofilm formation of *Aa* Δ*katA*. *Aa* VT1169 WT and Δ*katA* single species biofilms were cultured in CDM for 4 days, stained by STYO 9 and imaged by CLSM. The orthogonal images at the top and the 3D images at the bottom were shown **(A)** and biofilm biomass was quantified by COMSTAT **(B)**. Student's *t*-test. Means and standard deviations from triplicate experiments are shown.

Table S1 Strains used in this study.

Strain	Genotype and/or relevant characteristics	Source or reference
S. sanguinis SK36	Wild type, Human plaque isolate	[1]
P. gingivalis ATCC 33277	Wild type	ATCC
A. actinomycetemcomitans 652	Wild type	[2]
A. actinomycetemcomitans VT1169	Wild type	[3]
A. actinomycetemcomitans VT1169 Δ*katA*	VT1169, *katA* deletion mutant, Spc^R	[3]

Table S2 FISH probes used in the study

Name	Sequence (5'-3')	Fluorescent dye	Application
FISH-Ss	AGAGCAAGCTCCTCTCTTCAGCGTTC TA/3' atto 488/	ATTO 488	FISH staining of *S. sanguinis*
FISH-Aa	AGTACAAGTACTTACCTGCTACCGTC CGA/3' atto 590/	ATTO 590	FISH staining of *A. actinomycetemcomitans*
FISH-Pg	ACCTTAGCAAGCTAAGATCATGCTG CCCCT/3' atto 647N/	ATTO 647N	FISH staining of *P. gingivalis*

1 Kilian, M. & Holmgren, K. Ecology and nature of immunoglobulin A1 protease-producing streptococci in the human oral cavity and pharynx. *Infection and immunity* **31**, 868-873 (1981).

2 Brogan, J. M., Lally, E. T., Poulsen, K., Kilian, M. & Demuth, D. R. Regulation of Actinobacillus actinomycetemcomitans leukotoxin expression: analysis of the promoter regions of leukotoxic and minimally leukotoxic strains. *Infection and immunity* **62**, 501-508 (1994).

3 Ramsey, M. M. & Whiteley, M. Polymicrobial interactions stimulate resistance to host innate immunity through metabolite perception. *Proceedings of the National Academy of Sciences of the United States of America* **106**, 1578-1583, doi:10.1073/pnas.0809533106 (2009).
